# The Right to Know: *A Revised Standard for Reporting Incidental Findings*

**DOI:** 10.1002/hast.836

**Published:** 2018-03-28

**Authors:** G. Owen Schaefer, Julian Savulescu

## Abstract

*During the course of biomedical research, researchers sometimes obtain information on participants that is outside the aim of the study but may nonetheless be relevant to the participants. These incidental findings, as they are known, have been the focus of a substantial amount of discussion in the bioethics literature, and a consensus has begun to emerge about what researchers should do in light of the possibility of incidental findings. A consensus, however, is not necessarily correct. In this article, we address the common view that reporting of incidental findings should be based primarily on the possibility of medical benefit, factoring in the findings’ validity, clinical actionability, and significance to health or reproduction. While such medical beneficence should not be discarded, the need to give proper attention to participants’ autonomy, privacy, and interests (especially considering discussion of participants’ right not to know) suggests an alternative standard for when to report incidental findings: even if they are of no direct medical benefit, incidental findings should be reported based on the extent to which the participant can be expected to comprehend the information. We will offer a preliminary defense of this alternative as best respecting participants’ autonomy and privacy and promoting their interests. However, we acknowledge that the standard would face significant practical barriers, and these barriers lead us to* propose *a metaconsent addendum that would allow subjects to essentially waive the comprehension standard when resource or other constraints make meeting it impracticable*.

The “best‐medical‐interests” standard for reporting findings does not go far enough. Research subjects have a right to know about any comprehensible piece of information about them that is generated by research in which they are participating. An even broader standard may sometimes be appropriate: if subjects agree to accept information that they may not understand, then all information may be disclosed.

## Article

During the course of biomedical research, researchers sometimes obtain information on participants that is outside the aim of the study but may nonetheless be relevant to the participants. These incidental findings, as they are known, have been the focus of a substantial amount of discussion in the bioethics literature, and a consensus has begun to emerge about what researchers should do in light of the possibility of incidental findings. A consensus, however, is not necessarily correct. In this article, we address the common view that reporting of incidental findings should be based primarily on the possibility of medical benefit, factoring in the findings’ validity, clinical actionability, and significance to health or reproduction. While such medical beneficence should not be discarded, the need to give proper attention to participants’ autonomy, privacy, and interests (especially considering discussion of participants’ right *not* to know) suggests an alternative standard for when to report incidental findings: even if they are of no direct medical benefit, incidental findings should be reported based on the extent to which the participant can be expected to comprehend the information. We will offer a preliminary defense of this alternative as best respecting participants’ autonomy and privacy and promoting their interests. However, we acknowledge that the standard would face significant practical barriers, and these barriers lead us to propose a *metaconsent* addendum that would allow subjects to essentially waive the comprehension standard when resource or other constraints make meeting it impracticable.

Incidental findings can crop up in a number of ways. Researchers looking at the results of imaging studies might notice an abnormality that poses a health risk to the participant.^1^ Genetic screenings can reveal variants that are associated with higher relative risks of certain conditions.^2^ One such variant is the APOe4 genetic variant, which is strongly associated with earlier onset of Alzheimer's.^3^ And even a simple intergenerational study might reveal misattributed paternity.^4^ There are important differences between these examples concerning the salience of the information, its actionability, and other factors. Still, this discussion will present principles that should be applicable (even if the applications differ) in all contexts.

There are some obvious and uncontroversial strategies for dealing with incidental findings.^5^ Researchers should anticipate the possibility of incidental findings in advance of a study and form a plan for dealing with them. This plan will, in turn, be conveyed to research participants during the consent process, ensuring that participants are not completely blindsided if any incidental findings are revealed. We have no objection to these anodyne proposals, but they leave out the crucial detail of the content of the plan to deal with incidental findings. It might be useful to direct researchers to make some decision concerning incidental findings, but we can go further to evaluate the conditions under which incidental findings should be disclosed.

While recommendations about what incidental findings can and should be disclosed vary, there is a common thread, supported by a number of commentators, that could be described as the *best‐medical‐interests standard*.^6^ This standard tasks researchers with evaluating a given incidental finding along roughly three criteria: validity, significance to health (and sometimes reproduction), and clinical actionability. Validity refers to the accuracy and reliability of the finding; a mere suspicion of a problem would not merit reporting, but a finding informed by a number of clinical studies might. Significance to health and reproduction refers to whether the finding could have a substantial impact on someone's health or (via reproduction) that of his or her offspring. A life‐threatening brain tumor clearly passes this test, and a genetic variant related to earwax viscosity would not. And finally, clinical actionability refers to the potential for a clinical intervention to alleviate the health issue (or, perhaps, the possibility for the finding to alter reproductive decision‐making). Predispositions for treatable conditions like breast cancer would pass this test, while misattributed paternity likely would not (unless there was some expected medical intervention where paternity would be relevant). Commentators disagree on the relative weight and implications of each aspect of the standard, but they share the approach's tight focus on those three factors.

The best‐medical‐interests standard, however, is problematic because it is overly narrow. This narrowness comes from the clinical focus of the standard—health problems and clinical actionability. Participants may have significant interests in receiving incidental findings that are not related to their health or are health related but not actionable. For example, misattributed paternity would be of crucial interest to a purported father paying child support. And some people will have a nonmedical interest in learning about a genetic predisposition to currently incurable conditions like Alzheimer's on grounds that the knowledge can let them better plan their lives.

Still, there is some reason to think that dumping any and all incidental findings on participants is problematic and doesn't necessarily respect autonomy. We will ultimately argue for an alternative standard that allows for the disclosure of both clinically relevant information and information relevant to participants for reasons not directly related to health—though we will not quite recommend universal disclosure, instead proposing a novel comprehension standard. This argument relies on the notion of a person's right to know the details of incidental findings. There are a variety of ways to argue for the existence of such a right. We will be arguing primarily by analogy. The right not to know certain information is widely debated in the literature on incidental findings—but, we will show, arguments for the right not to know are grounded in commitments that imply a corollary right to know, at least under certain circumstances.

Our arguments are aimed primarily at those who accept the right not to know. They may therefore not be convincing to those who deny the existence of such a right. But while addressing such positions is outside the scope of this paper, we will discuss later that both defenders and critics of the right to know rely on a concept of autonomy that supports the right to know.

## The Right Not to Know and Its Implications

In the discussion of incurable conditions like Alzheimer and Huntington diseases, much concern has been expressed about the right of individuals not to receive certain information. When faced with unavoidable mental decline or premature death, some would prefer to remain in blissful ignorance of their fate. Yet a well‐meaning physician or researcher with knowledge of an incurable diagnosis might be uncomfortable withholding it; the information is crucially relevant to the individual's health, after all, even if there is no medical treatment for the disease. The question then arises whether individuals have a right not to know certain information about themselves—or, in the present context, whether they have the right to refuse certain incidental findings. The usual answer in the literature is that there is indeed a right not to know information about oneself. But that stance is typically grounded in ways that also imply a converse right—the right to know information about oneself.

### Interests

There are several arguments defending the right not to know. These are grounded in interests, privacy, and (most commonly) autonomy. We will address each in turn. On the interests account, individuals have a right to decline information about themselves based on personal interests that might be set back by receiving that information. Here, we might understand the term “right” loosely, as simply reflecting the moral imperative not to set back someone's interests via the disclosure of information. Some information, like a fatal diagnosis, may well set back someone's interests, by causing stress and anxiety, for example, or by creating problems for obtaining insurance.^7^ The interests account only motivates a provisional right not to know; in some cases, the individual's preferences will be overridden by satisfaction of other interests, like health and safety. Still, there is good reason to take preferences seriously when evaluating the effects of information on interests. Individuals will often be in a particularly good position to evaluate whether some piece of information will indeed set back their interests. They have firsthand knowledge of their own experiences, dispositions, goals, values, and so on that will be crucial in evaluating whether their interests have indeed been set back. Physicians or researchers may sometimes have greater knowledge of, say, the typical likelihood or severity of anxiety that the information causes, but that knowledge could be conveyed to participants to make them better‐informed in their evaluation of whether disclosure would set back their interests.

But if the provision of information can sometimes set back interests, so too can the lack of information. Uncertainty, too, can cause anxiety, and information has general utility in helping people carry out their personal goals and priorities. For example, overestimation of one's likelihood of developing dementia could cause unwarranted fear, while underestimation could cause insufficient planning for care and support at older ages. In addition, subjects could interpret the lack of results as a positive sign about their health—“no news is good news,” as one study interviewing recipients of genetic testing found.^8^ Some of this could be ameliorated by more effective communication strategies when obtaining consent, emphasizing that an absence of reported findings would not imply that the individual is in good health. But the opportunity costs remain, since failure to disclose a finding prevents a patient or subject from learning something important about themselves and potentially taking action in response. And once again, personal preferences will be a decent guide to whether provision of the information would indeed be in someone's interests. If a setback of interests grounds the right not to know in certain circumstances, then it should also ground a right to know.

This outcome might appear to be in line with the best‐medical‐interests standard laid out above. However, crucially, the present account is not limited in scope to medical interests; other interests—personal goals, relationships, access to insurance, or simple morbid curiosity—can also count. The debate over the right not to know has already appropriately moved beyond mere medical interests. And in such areas, unlike the medical context, individuals will be in a much better place to determine whether the information is relevant and should be disclosed compared to a researcher.

### Privacy

Graeme Laurie has defended an alternative privacy account of the right not to know. Laurie identifies a basic right “concerned with the control of personal information and with preventing access to that information by others.”^9^ Most obviously, this right would disallow researchers from analyzing incidental findings without the consent of the participant. This leads to a right against disclosure of unwanted information in the research context: if researchers are not allowed to attend to incidental findings, then they are not allowed to report them. Yet Laurie suggests that the right protects more than just access to personal information; it affords individuals control over that information. The idea is that the intensely personal nature of the information and its relation to the self gives people special authority over it. People have a right over how they engage with the world, including engaging with their own personal information. This would include deciding to learn less about oneself, grounding a right not to know personal information.

Laurie's understanding of privacy certainly also implies a right to know personal information. The right to privacy, as Laurie understands it, is not a purely negative guard against unwanted use of information. The notion of control has a positive connotation as well. Just as the right to control one's property implies the right to decide to whom one's property should be transferred, the right to control one's personal information implies the right to decide with whom that information should be shared—including the agent herself. A researcher who denied incidental findings to a participant would be taking control over personal information out of the hands of the participant, preventing them from engaging with the world as they see fit. Participants would have a right to know incidental findings, on this view, stemming from that prerogative to control the flow of personal information.

### Autonomy

While Laurie offered the privacy account as an alternative to the more dominant autonomy account, it resembles that account in its emphasis on control. Autonomy, after all, is literally self‐governance—not far from the notion of having control over oneself. Roberto Andorno argues, for example, that disclosure of unwanted information violates people's autonomy by taking informational decision‐making out of the hands of the subject of the information and putting it in the hands of a third party (such as a researcher).^10^ The third party might justify failure to disclose based on harms, but then we would have a paradigmatic form of paternalism—violating someone's autonomy in order to further the person's interests. Furthermore, forcing information on people in effect limits their options (shutting off their ability to choose between ignorance and knowledge) and, on some accounts, would be a form of coercion.^11^

Broad views of the autonomy‐based right not to know will imply a corresponding right to know personal information. Joel Anderson and Warren Lux, for instance, identify alienation as inimical to autonomy.^12^ To act autonomously, one needs a tight connection between one's intentions and one's actions; this will involve, among other things, an accurate self‐assessment of one's dispositions, capacities, and other features.^13^ Acting without self‐understanding makes one's actions less one's own.

The right not to know might seem incompatible with this view of alienation, since ignorance conflicts with accurate self‐assessment.^14^ However, the difficulty may be avoided by taking a higher‐order view of alienation; while willful ignorance may undermine accurate self‐evaluation, it may not lead to alienation if one is intentionally embracing that ignorance. One can make sense of one's actions, even when they fail, because one has endorsed the (ignorant) process by which they came to be. In any event, the emphasis on control over personal information contained in broader views like this lends support to the right to know personal information. Researchers who refrain from giving incidental findings (especially those that would inform particular choices) facilitate someone's inaccurate self‐assessment, potentially leading to alienation and inhibiting them from acting in a fully autonomous manner.

Opponents of the right not to know also rely on the notion of autonomy—on grounds that it is essentially incompatible with ignorance.^15^ To act autonomously, on this picture, is to make informed decisions. Having more information should, after all, make one more able to govern oneself. Information allows one to properly take various factors into account, predict the future more accurately, weigh up potential consequences properly, and so on. In the case of a fatal diagnosis, the revelation may cause stress, but it will also allow one to avoid making long‐term plans that could never be fulfilled or to prioritize important projects one would otherwise put off.

This suggests that there is an intimate connection between information and autonomy. Providing incidental findings to participants would allow them to better govern their lives and, in that way, to promote their autonomy. There are two ways this connection could in turn support a right to know personal information. First, one might have a right to reciprocal assistance (here, in the form of autonomy promotion) in the context of research. One has allowed oneself to be subjected to experimentation for the sake of others, and it would be ungrateful for researchers to refuse to promote individuals’ autonomy via the disclosure of incidental findings.^16^ It might be argued that there are other means for researchers to show their appreciation to subjects. However, these means are limited (due to concerns about undue inducement, compensation is often capped), and moreover, there is a sense in which reciprocity makes the most sense when it is in kind—that is to say, when researchers repay the knowledge‐based benefit that subjects have given them by providing a knowledge‐based benefit to subjects. Returning overall study results is another way of accomplishing that, but many subjects will probably find individual incidental findings of more interest than general study results.

And second, there is a sense in which the failure to disclose the information is not merely a failure to promote autonomy but is in fact equivalent to active disrespect of autonomy. Respect for autonomy involves putting decision‐making power in the hands of the individual affected by a decision, rather than giving it to third parties. Withholding information takes that power away from individuals and puts it in the hands of third parties (or no one at all), thus potentially violating their autonomy.

## A Comprehension Standard for Disclosure

The preceding section should provide some motivation for a right to know personal information, and in the research context, this right would extend to incidental findings. While this view might not be popular among researchers themselves,^17^ it will likely be welcomed by research participants. One 2006 survey found that, among 105 neuroimaging study participants, 97 percent wished to have incidental findings of incurable malignancies disclosed, and 91 percent wanted even benign abnormalities reported.^18^ Other more recent studies have found similar support for reporting genetic incidental findings.^19^ Even when individuals are reluctant to hear results, they want to be given control over whether results will be disclosed.^20^ This indicates significant support among subjects for the right to know incidental findings. The existence of such preferences shows that there is at least subjective value in receiving incidental findings. That is, by the lights of potential recipients, it advances their interests. We should take these perspectives seriously, given that such individuals are in a unique first‐person position to evaluate their own values and interests. And these perspectives are not arbitrary or irrational—as seen above, we can elucidate sound ethical bases for respecting them.

Various commentators acknowledge the significance of participant preference,^21^ but what exactly does that imply? Universal disclosure of incidental findings would be the most natural implication, but we must be careful. We have, so far, been defending the right to know personal information, and knowledge implies genuinely comprehending something. Merely *hearing* information certainly does not ensure comprehension, and the complex nature of some incidental findings might make understanding difficult.

The right to know personal information may therefore lend support to a defeasible duty on the part of researchers to help participants understand any incidental findings. Reports may be in overly technical jargon, or be couched in difficult‐to‐understand statistical terms, or have unclear implications for one's life, and so on. Even the choice of whether to receive incidental findings in the first place is affected by misunderstandings, variable moods, framing effects, and other problematic factors.^22^ Researchers should in the very least be prepared not just to state findings but to explain them in a way that could best assist in helping the participant understand them. In the area of genetic research, that may require some genetic counseling (or assistance in obtaining it).^23^

Yet even with such assistance (and especially given cost constraints, as discussed below), many individuals will still misunderstand the content of a given incidental finding. Participants lack the training and expertise of researchers, and brief explanations can go only so far in helping them understand a given finding. So suppose there is an incidental finding concerning a rare genetic mutation associated with a complex pathology—one so complex that researchers can accurately and reliably predict that the participant would misunderstand the mutation, significantly overestimating the likelihood and effect of developing the pathology. Does one have a right to receive information that one will misunderstand?

It would be difficult to defend such a right based on autonomy considerations. Recall that autonomy is relevant for disclosure because an accurate self‐assessment is central to autonomy. But autonomous self‐governance would hardly be promoted by an inaccurate or distorted understanding. Having a deeply misinformed understanding of a mutation will in turn lead to actions that are misinformed and based on ideas that do not match reality. Participants would have a worse understanding of themselves, be less able to direct their actions or achieve their goals, and be unreliable in shifting priorities in light of the information.

Similarly, Laurie's privacy concerns would not be affected by failing to disclose information likely to be misunderstood. At the center of this notion of privacy is one's self; information is to be under one's control due to its intimate relation to that self, a domain over which the individual has special rights. But misunderstood information is not at all related to that sense of self; in fact, misinterpreted information runs the serious risk of fostering a flawed appreciation of who one is. Participants might overestimate the risks associated with a finding, thinking themselves doomed when that is far from the case. Or they might be lulled into a sense of complacency by underestimating risks and think themselves safe from a very real threat. Either way, provision of the information would contribute to a distorted rather than enlightened understanding of oneself and therefore would not be required by respect for privacy.

It is more plausible, though, to think that someone's interests could be advanced even by information they misunderstand. Consider a medically actionable incidental finding; the participant might misinterpret what's going on, but the information could be passed on to a medical specialist who's in a position to recommend a treatment that the participant would greatly appreciate. For this reason, information that passes the standard best‐medical‐interests test should be reported even in the case of ignorance. But we would still need to know what should be done in circumstances where an incidental finding fails that test—where researchers do not think there is a serious medical condition that could be treated by some intervention.

In such circumstances, we recommend that incidental findings be disclosed based on participants’ likelihood of misunderstanding them. This standard respects the right to know (whether based in autonomy, privacy, or interests) by helping ensure that a participant actually comes to know the relevant finding. It allows disclosure of findings that participants care about outside the medical context. Researchers need not impose their own values on subjects; they would, in essence, be deferring to participants’ judgments in deciding whether to care about and how to deploy a given finding. Most of all, the standard facilitates subjects’ control over their lives by empowering them with greater understanding of their biological states.

We should be careful not to take this principle too far. A determination that patients will process information improperly may appear overly paternalistic. Practitioners would be asserting epistemic superiority over subjects and exerting a level of control over their lives that could appear disrespectful. For this reason, the bar for nondisclosure would be significant. Researchers must have good reason based on sound empirical data—not merely a suspicion—that a significant misunderstanding would occur. Failure to comprehend the medical implications is not enough. A participant's epistemic situation must be made significantly worse; they must be expected to have a worse understanding of their condition than they otherwise would. For example, suppose a genetic finding suggested a doubled likelihood of development of a rare debilitating condition, from a 0.5 percent likelihood to a 1 percent likelihood. If participants would be likely to focus on the “doubled” aspect and think they are now more likely than not to develop their condition (implying a greater than 50 percent chance of developing the condition), then we can safely say the information provision would significantly distort their state of mind. This is because the information would lead them to overestimate their chances of developing the condition by a factor of fifty.

It is quite possible that the number of individually comprehensible incidental findings could be very large for genomic studies. A large data dump might not serve subjects’ interests very well. However, such a large dump could in aggregate itself be subject to misunderstanding. Because it is an aggregate, sorting the information for subjects may be required. These sorting mechanisms might be along the lines of the more dominant models currently available, focusing first on the most clinically significant and going down the line. But it still would leave adequate room for individual discretion, insofar as individuals can make judgments on their own as to the relevance of the information provided—assuming it is presented in a comprehensible format.

Which findings would end up passing the further comprehension test would be the subject of further inquiry, and outside the scope of this article. We can suggest, however, the sort of considerations that would weigh heavily. There is well‐known evidence that people overestimate low probabilities.^24^ And many incidental findings, especially in genetics, will involve either a low absolute level of risk or a small but statistically significant increase in relative risk. If people systematically misinterpret such results by significantly overestimating their likelihood of receiving a condition, then the finding would fail the comprehension test (as in the above example). The information would distort rather than improve their decision‐making processes (though it may still need to be disclosed based on the best‐medical‐interests standard).

By contrast, other sorts of incidental findings will be clearly and distinctly understood. Misattributed paternity may be a paradigmatic case of an incidental finding that would pass the comprehension test. The notion of biological parentage is not incredibly complex and can be easily grasped by a participant without any medical training. It is also the sort of information that many individuals would want and that may inform a large number of personal decisions concerning one's family. For this reason, misattribution of paternity would pass the comprehension test; even when there is no clear medical benefit to the information, researchers have good reason to disclose it.

Amulya Mandava, Joseph Millum, and Benjamin Berkman have argued that, on the contrary, misattributed paternity should generally not be disclosed due to the potential harms and an asymmetric duty to avoid harming compared to providing benefit.^25^ They are thus operating on the “interests” framework identified above. To be sure, in some circumstances, potential harms may outweigh subjects’ right to know their paternity. However, in their analysis of harms and benefits, Mandava, Millum, and Berkman focus on psychosocial harms such as distress, breakdown in familial relations, and retaliation, while comparing them to clinical benefits. But disclosure may also prevent psychosocial harms, such as maintaining a deeply mistaken understanding of one's family and a relationship that is based on deceit. Reports of misattributed paternity may cause familial breakdowns precisely because people place a great deal of importance on valid paternity for their relationships. In any case, the best way for researchers to address this is not to hide the information, but to be up front at the beginning of a study involving multiple family members—offering the option to receive (or not) a report of misattributed paternity should it arise. It is more respectful to let subjects judge for themselves whether the report would cause them undue harm than for researchers to make paternalistic judgments about the relative value of the information. (In theory, informing participants of the possibility of a misattributed paternity during the consent process could itself cause distress. But the potential for distress to result from the consent process exists with respect to many risks disclosed during it, such as the risk of death for certain surgical studies; it is generally accepted that such risks should nevertheless be disclosed.)

It may be that some potential recipients, due to differing educational backgrounds or other factors, may be more or less able to comprehend a given finding. Nevertheless, those reporting findings should adopt a general standard that applies to all mentally competent subjects. To do otherwise risks unfairly withholding information from certain disadvantaged groups while providing it to more advantaged groups.

The practicalities of determining which findings are comprehensible and which are not may prove too difficult for individual researchers. To address this, it is helpful to consider the approach taken by the American College of Medical Genetics and Genomics, which curates a list of reportable genetic variants based on the best‐medical‐interests test.^26^ While that list has engendered controversy, it is also very useful to individual researchers, who are ill‐equipped to make adequate judgments about the significance of individual findings. A similar list, but based on the comprehension standard, could be generated by scientists in collaboration with psychologists. That list could then be used to determine which incidental findings are to be reported.

The list would of necessity be guided by the general comprehensibility of findings. Lack of sensitivity to individual variation may compromise our ability to guarantee that a given recipient understands the information provided. Nevertheless, a general standard concerning which findings to return is entirely appropriate, as it promotes fairness in information provision and ensures that some privileged few do not have access to greater information. Recipient background and other factors may, however, reasonably affect the way findings are communicated and explained (particularly by genetic counselors).

What we have, then, is something less than a standard of universal disclosure.^27^ It's a two‐stage test for disclosure of incidental findings: First, determine whether the finding passes the best‐medical‐interests test. If it does, disclose the information to the subject; if it does not, apply a second comprehension test. If it passes that test, disclose it, and if it fails, do not disclose it. By adding a second test only after the first test's failure, our approach is more permissive than many current recommendations—but it does not open the floodgates to having all sorts of erroneous information given to participants. Rather, it respects the right of participants to know incidental findings without imposing on them information that would in fact end up inhibiting self‐knowledge.

## Metaconsent

While researchers would ideally promote subjects’ comprehension of incidental findings, in reality, they are likely to face such significant difficulties that the goal could sometimes be impracticable. Often, they are not trained in providing such information, and their resources may be limited. One response would be to pull back on the scope of incidental findings reported. However, as we have argued for a right to know the content of incidental findings, we will propose an alternative: a form of metaconsent, through which participants would have the opportunity to explicitly accept information they may misunderstand.

Thomas Ploug and Søren Holm have proposed metaconsent in the context of future use of one's research data. In their proposal, subjects would select, based on the type of research, what sort of consent they would like to receive (whether detailed, broad, blanket allowance, or blanket refusal) for any future research with their data.^28^ The metaconsent is intended to respect their higher‐order preferences concerning what level of consent is itself appropriate, tailoring the consent process to the unique values of each subject.

Here, we adapt the notion of metaconsent to the context of incidental findings. At the point of initial consent, participants would be made aware not only of the potential for incidental findings but also that the findings could be complex and difficult to understand and that, due to practical or budgetary constraints, the researchers will be unable to provide further explanation or counseling concerning the information. Participants could then decide whether to receive such findings sans context or counseling, relieving researchers of the duty to spend resources analyzing or explaining it.

This approach may seem to be incompatible with our earlier emphasis that, when information is not medically actionable, respecting the right to know does not require disclosing information that would be misunderstood. However, offering the information might be justified on the basis of respect for autonomous metacognition. By metacognition, we mean control over the conditions that inform cognition itself. Some participants might be more willing than others to accept the cognitive risk that they would not properly understand some finding, and this provision would allow them to decide for themselves whether that risk is worthwhile.

In this way, metaconsent would be a means to put control of personal knowledge squarely in the hands of participants. The approach avoids some of the paternalistic pitfalls of the more moderate comprehension standard insofar as it is participants rather than researchers who would determine whether potential misunderstanding is worthwhile. And in doing so, it allows participants to make a personal risk assessment more sensitive to their individual interests. To be sure, it may involve some trade‐off. If misunderstanding is inimical to autonomy, then it may be the case that metaconsent is tantamount to giving participants the autonomy to limit their autonomy. Whether this is acceptable will depend on whether control or comprehension is more central to autonomous decision‐making.

Allowing a waiver of explanation of incidental findings would undoubtedly be controversial. It would differ from more anodyne proposals to communicate one's disclosure plan at the outset of a study insofar as metaconsent requires offering disclosure of incidental findings sans institutional support like genetic counseling while also (unlike blanket reporting proposals) requiring that the risks of misinformation be disclosed. Metaconsent could reasonably be seen as a threat to informed consent more generally—if people can waive proper explanation in the context of incidental findings, then why not also allow subjects to waive proper explanation of a study's purpose, side effects, benefits, and so on? But we could draw a distinction based on the nature of the information. Metaconsent concerns the provision of information concerning one's biological state, not concerning something that is about to happen to someone's body—or even information to be received by the researchers. There may still be good reason to require understanding of what is involved when one subjects oneself to experimentation. That said, informed consent may not require full comprehension of the nature and purpose of a study.^29^ Metaconsent may be consistent with a more limited comprehension standard that nevertheless respects autonomy. There is not space here to fully explore these issues, but they warrant further consideration.

## Objections

### Psychological harm

So far, we have mostly been discussing the positive reasons to provide incidental findings to participants—improving their autonomy, respecting their privacy, advancing their interests. But a number of commentators have pointed out that information can sometimes be psychologically harmful. It may induce anxiety, fear, survivor's guilt, depression, or other negative mental states.^30^ Even if there is a right to know, it may not be absolute, and the negative effects of some findings (especially those that are not actionable) may override that right. Researchers may feel there is a duty to avoid harming participants, even if that means depriving them of information they want.

This objection relies on somewhat questionable empirical premises. Some studies on disclosure of genetic findings of disposition toward Huntington disease and breast cancer indicate that, while disclosure may have some initial negative effects, the effects are not long‐lasting, and people eventually return to normal levels of anxiety and stress.^31^ Conceivably, other findings have more long‐lasting negative effects, but no evidence yet supports that possibility. For the time being, the decision about disclosure involves weighing a certain positive reason to provide information (respecting the right to know) against an uncertain negative reason against it (psychological harm). In such circumstances, we would be justified in siding with the certain benefit, especially when current evidence indicates the uncertain negative harm would not be extreme or long term.

Even if there were evidence that disclosure of incidental findings caused severe harm, a blanket ban on the provision of such information would not be the most respectful response. Rather, researchers should inform participants of that very risk, at the point of initial consent, and allow them to decide for themselves whether it is worth the trouble. This approach has the advantage of avoiding the paternalistic imposition of values and allowing researchers to avoid the difficult weighing‐up of the importance of information against psychological harms. Assuming they could properly understand the information on the potential harms, participants would be in a better position than researchers to appreciate whether the information was worth it.

### Resources

Because our proposal recommends providing more incidental findings than the standard best‐medical‐interests test on its own, it may be more costly than other approaches. Analyzing an incidental finding to ensure that it is accurate and reliable and then reporting it are not at all insignificant steps; they take time and resources that could otherwise be spent on the research itself. We need to weigh up the importance of respecting the right to know against the effects on the study itself.^32^

At the extreme, it may be that providing incidental findings that fail the best‐medical‐interests test would make the study too costly to carry out. In such a case, going ahead with the study without providing the findings would be acceptable. This is so because, under the circumstances, there is no practical way to provide participants with knowledge of the incidental findings; the attempt to respect their right to the information would be self‐defeating because no such information would be gained at all.

But what about the more likely scenario, where a study could go on with our revised standard but would take longer or produce weaker results because of a more limited budget? A complete answer requires determining whether respect for participants’ right to know is more important than the marginal benefit of a more efficient study. When the provision of incidental findings requires fewer resources because it is easily analyzed and conveyed, then the responsibility to disclose will be quite substantial. Misattributed paternity is a good contender for such low‐cost information because it is easy for participants to understand. Genetic information that requires substantial counseling is more difficult and must be evaluated case by case. As a rule of thumb, the more significant the information, the more funds that should be spent to deliver it accurately and without misunderstanding.

Arguably, the costs of counseling and related services should not be borne by researchers but the state. This will make the most sense in countries with a universal health service whose duty it is to provide care. In the United States, however, specific policies for funding may need to be developed and integrated into the scope of mandated health coverage. And when external bodies are unable or unwilling to pay for counseling, the question then falls to researchers. Unfortunately, until there has been systematic reform, researchers may have to bear the costs of ensuring that information is understood. That is what it is to respect participants as persons—but again, this duty is not unlimited and will vary depending on the costs of promoting comprehension.

In any case, the metaconsent modification to the comprehension standard should limit the circumstances in which cost constraints truly prevent the reporting of incidental findings. Allowing individuals to consent to receive bare‐bones incidental findings reports would save time and resources. At the same time, we should encourage researchers and funders, where possible, to make further resources available to provide proper information and context; metaconsent should be invoked only when costs are truly an issue, and not as a default option.

While researchers may have a primary responsibility to create generalizable knowledge, they also have ancillary duties to protect participants’ autonomy, privacy, and interests. Our proposal for when to disclose incidental findings is sensitive to all these considerations. Findings that are in the best medical interests of participants should generally be disclosed, but proper attention to participants’ right to know personal information suggests that researchers have strong reason to disclose findings even when they are not clearly in the participants’ best medical interests. Disclosure should by default be limited to circumstances when (after appropriate explanation) participants would actually understand the information, so incidental findings need not always be disclosed. Moreover, given practical challenges in ensuring full understanding of results, we suggest that, for some research, participants could provide metaconsent to receive incidental findings that they know they might misunderstand.

This proposal supports the reporting of a wider range of incidental findings than is commonly recommended. Personal information should generally be under the purview of the individual whom it concerns, and the overall goal in disclosure should be to map out a reasonable way for researchers to respect people's right to know about themselves.
